# Prevalence and Clonal Distribution of *pcpA*, *psrP* and Pilus-1 among Pediatric Isolates of *Streptococcus pneumoniae*

**DOI:** 10.1371/journal.pone.0041587

**Published:** 2012-07-25

**Authors:** Laura Selva, Pilar Ciruela, Krystle Blanchette, Eva del Amo, Roman Pallares, Carlos J. Orihuela, Carmen Muñoz-Almagro

**Affiliations:** 1 Molecular Microbiology Department, University Hospital Sant Joan de Deu, Barcelona, Spain; 2 General Directorate of Public Health, Government of Catalonia, Spain; 3 Department of Microbiology and Immunology, The University of Texas Health Science Center at San Antonio, San Antonio, Texas, United States of America; 4 Department of Infectious Diseases, Idibell, Ciberes, Hospital Bellvitge, University of Barcelona, L’Hospitalet, Barcelona, Spain; Columbia University, United States of America

## Abstract

*Streptococcus pneumoniae* is the leading cause of vaccine-preventable deaths globally. The objective of this study was to determine the distribution and clonal type variability of three potential vaccine antigens: Pneumococcal serine-rich repeat protein (PsrP), Pilus-1, and Pneumococcal choline binding protein A (PcpA) among pneumococcal isolates from children with invasive pneumococcal disease and healthy nasopharyngeal carriers. We studied by Real-Time PCR a total of 458 invasive pneumococcal isolates and 89 nasopharyngeal pneumococcal isolates among children (total = 547 strains) collected in Barcelona, Spain, from January 2004 to July 2010. *pcpA*, *psrP* and pilus-1 were detected in 92.8%, 51.7% and 14.4% of invasive isolates and in 92.1%, 48.3% and 18% of carrier isolates, respectively. Within individual serotypes the prevalence of *psrP* and pilus-1 was highly dependent on the clonal type. *pcpA* was highly prevalent in all strains with the exception of those belonging to serotype 3 (33.3% in serotype 3 isolates vs. 95.1% in other serotypes; P<.001). *psrP* was significantly more frequent in those serotypes that are less apt to be detected in carriage than in disease; 58.7% vs. 39.1% P<.001. Antibiotic resistance was associated with the presence of pilus-1 and showed a negative correlation with *psrP*. These results indicate that PcpA, and subsequently Psrp and Pilus-1 together might be good candidates to be used in a next-generation of multivalent pneumococcal protein vaccine.

## Introduction

Invasive disease caused by *Streptococcus pneumoniae* is responsible for more than 1.6 million childhood deaths worldwide every year [1]. In certain developed countries, including Spain, despite vaccination with a 7-valent conjugate vaccine against capsular polysaccharide (PCV-7), pneumococcal pneumonia remains a major cause of pediatric hospital admission [2], [3], [4]. PCV-7 is composed of capsular polysaccharide from serotypes 4, 6B, 9V, 14, 18C, 19F, and 23F individually conjugated to diphtheria CRM197 and has proved to be effective in preventing pneumococcal disease caused by these serotypes in children [5]. PCV7 also prevents invasive pneumococcal disease (IPD) in adult and non-vaccinated children by an indirect effect (herd immunity) on pneumococcal transmission [5], [6]. Importantly, nowadays evidence exists of the emergence of non-vaccine serotypes in children and adults to occupy this vaccine-emptied niche, thereby partially eroding the benefit of PCV-7 [3], [7], [8], [9]. For example, in Spain disease caused by serotype 19A was responsible for 13.5% of pediatric IPD during the period 2000–2008, whereas in 2000, at the time of introduction of PCV-7, serotype 19A only accounted for 4.6% of pediatric infections [10]. The pneumococcus is also a primary cause of otitis media and PCV-7 only slightly reduces the rate of disease [11]. At present, more than 1,500,000 cases occur annually in the United States, with an estimated cost of 440 million U.S. dollars [12]. Thus, pneumococcal disease remains a major medical problem with an urgent need for an improved vaccine.

Due to these limitations, other conjugate vaccines with a larger number of serotypes have been recently commercialized. These include a 10-valent conjugate vaccine (PCV10), which includes the seven serotypes of PCV7 plus serotypes 1, 5 and 7F and PCV13 (PCV10 plus additional serotypes 3, 6A and 19A). These vaccines will most likely continue to reduce the burden of invasive pneumococcal disease and are becoming increasingly available in underdeveloped countries due to efforts of institutions such as The Bill and Melinda Gates Foundation through GAVI Alliance [13], [14]. However, due to the high cost of the conjugation process, these vaccines are limited in the number of serotypes that can be included in an affordable vaccine. The current cost for each dose of PCV13 is $100–125, with three immunizations recommended.

An alternate vaccine strategy is the use of a serotype-independent vaccine using conserved common pneumococcal protein antigens. These might stand alone, or replace the diphtheria toxoid in the conjugate vaccine and thereby enhance coverage of the existing vaccines. To date, numerous preclinical studies have shown that different pneumococcal proteins confer protection against pneumococcal challenge and that a combination of multiple proteins confers superior protection. The main advantage of a protein vaccine is that protection would not be serotype dependent and fewer antigen candidates could offer a high coverage with a lower cost of manufacturing. For these reasons, studies are warranted in determining if a next-generation of a multivalent protein vaccine against pneumococcus is feasible and desirable.

The objective of the present study was to determine the distribution and clonal type variability of three novel potential vaccine candidates: Pneumococcal serine-rich protein (PsrP), Pilus-1, and Pneumococcal choline binding protein A (PcpA). PsrP is a serine rich repeat protein (SRRP) previously demonstrated to be responsible for lung-cell attachment and *in vivo* biofilm formation [15], [16]. Pilus is a long organelle that, like PsrP, extends beyond the polysaccharide capsule and acts as an adhesin [17]. Finally, PcpA is a choline-binding protein with a role in pneumococcal adhesion and biofilm formation [18], [19]. Determining the prevalence and distribution of these proteins in strains that cause IPD and their correlation with disease and antibiotic resistance could be of great value for future vaccine formulations.

## Methods

### Clinical Isolates

All pediatric invasive pneumococcal isolates characterized by the Molecular Microbiology Department at University Hospital Sant Joan de Deu in Barcelona, Spain from January 2004 to December 2010 were included in this study. The department performs molecular surveillance of pneumococci in Catalonia, Spain. Clinical isolates were obtained from patients admitted to Sant Joan de Déu Hospital and, since 2009, from patients attended in 30 health centers throughout Catalonia region. In addition, we also included eighty-nine pneumococcal strains isolated from nasopharynx of healthy children during 2004–2008.

### Serotyping and Antimicrobial Susceptibility

All isolates were serotyped by Quellung reaction at the National Pneumococcus Reference Centre (Majadahonda, Madrid). Pneumococcal isolates collected since 2009 were also serotyped by Real-Time PCR (RT-PCR) using published protocols [20]. Serotypes were classified according to coverage of the existing 7,10, and 13-valent conjugate vaccines and their attack rate according to the studies of Brueggemann *et al.* [21] and Sleeman *et al.* [22]. Serotypes with high attack rate (those that are less apt to be detected in carriage than in disease) included: 1, 4, 5, 7F, 9V, 14, 18C and 19A. Serotypes with low attack rate (that are less apt to be detected in disease than in carriage) included: 3, 6A, 6B, 8, 9N, 10A, 11A, 12F, 13, 15A, 15BC, 16F, 17F, 19F, 20, 21, 22F, 23A, 23B, 23F, 24F, 27, 31, 33F, 35B, 35F, 37 and 38. Agar dilution technique was used to determine the minimal inhibitory concentrations (MICs) of penicillin and other antibiotics. Antibiotic susceptibility was defined according to the 2008 meningeal breakpoints established by the Clinical Laboratory Standards Institute [23]. Isolates with intermediate or high level resistance were defined as non-susceptible.

### Extraction of DNA

Genomic DNA was extracted from bacteria using Chelex-100 resin (BioRad Laboratories, Hercules, California, USA). Briefly, pneumococci scraped from blood agar plates were suspended in 100 µl of PBS-buffer; 50 µl were transferred to a new microcentrifuge tube and vigorously vortexed with 150 µl of 20% w/v Chelex-100 in PBS. The bacteria/resin suspensions were incubated for 20 minutes at 56°C followed by a 10-minute incubation at 100°C. After cooling and centrifugation, the supernatant was used as a DNA template in PCR reactions.

### Multilocus Sequence Typing (MLST)

Genetic characterization of pneumococci was performed using MLST. In brief, internal fragments of the *aroE*, *gdh*, *gki*, *recP*, *spi*, *xpt* and *ddl* genes were amplified by PCR using the primer pairs described by Enright and Spratt [24]. PCR products were sequenced using an ABI 3130xl GeneticAnalyzer (Applied Biosystems). The sequences at each of the seven loci were then compared with all of the known alleles at that locus. Sequences that are identical to a known allele were assigned the same allele number whereas those that differ from any known allele were assigned new allele numbers. The assignment of alleles at each locus was carried out using the software at the pneumococcal web page: www.mlst.net. The alleles at each of the seven loci define the allelic profile of each isolate and their sequence type (ST). Allelic profiles are shown as the combination of 7 alleles in the order *aroE, gdh, gki, recP, spi, xpt and ddl*. A clone is defined as a group of isolates with identical allelic profile or ST.

### Real-Time PCR Assay

We analyzed the nucleotide sequence of *psrP,* pilus-1 subunit *rrgC*, and *pcpA* for primers in all publically available *S. pneumoniae* genomes available through the United States National Center for Biotechnology Information web site (http://www.ncbi.nlm.nih.gov/). The primers and probe selected for *psrP* detection were: forward primer: 5′-CTTTACATTTACCCCTTACGCTGCTA; reverse primer 3′ CTGAGAGTGACTTAGACTGTGAAAGTG and probe: FAM-CTGGTCGTGCTAGATTC (Quencher MGB). These primers identified a conserved region within Basic Region domain of PsrP. For pilus-1 detection the primers and probe were: forward primer: 5′-TTGTGACAAATCTTCCTCTTGGGA; reverse primer: 3′-GTCACCAGCTGATGATCTACCA and probe: FAM-CAGTGGCTCCACCTCC (Quencher MGB). These primers identified a conserved region within the structural subunit protein RrgC encoded in the *rlrA* islet of pilus type 1. For *pcpA* detection the primers and probe were: forward primer: 5′-GAAAAAGTAGATAATATAAAACAAGAAACTGATGTAGCTAAA; reverse primer: 3′-ACCTTTGTCTTTAACCCAACCAACT and probe: FAM-CTCCCTGATTAGAATTC (Quencher MGB). These primers identified a conserved region of N-terminal fragment of PcpA. Finally, as a positive control and to test PCR inhibitors and DNA quality, detection of *ply* gene by Real-Time PCR was performed as previously described in all strains [25]. *Ply* encodes the pneumolysin, a toxin found within all *S.pneumoniae.*

The reaction volume for each gene detected was a total of 25µl and contained 5µl of DNA extract from samples or controls and 12.5µl 2X TaqMan Universal Master Mix (Applied Biosystems), which includes dUTP and uracil-N-glycosylase; each primer was used at a final concentration of 900 nM. The TaqMan probes were used at a final concentration of 250 nM. DNA Amplification was done performing universal amplification conditions: incubation for 2 min at 50°C (uracil-N-glycosylase digestion) and 10 min denaturation at 95°C, 45 cycles of two-step amplification (15 s at 95°C, 60 s at 60°C). Amplification data were analyzed by SDS software (Applied Biosystems). The reporter dye was measured relative to the internal reference dye (ROX) signal to normalize for non-PCR related fluorescence fluctuations occurring from well to well. The cycle threshold (CT) value was defined as the cycle at which the reporting dye fluorescence first exceeds the background level.

**Figure 1:**
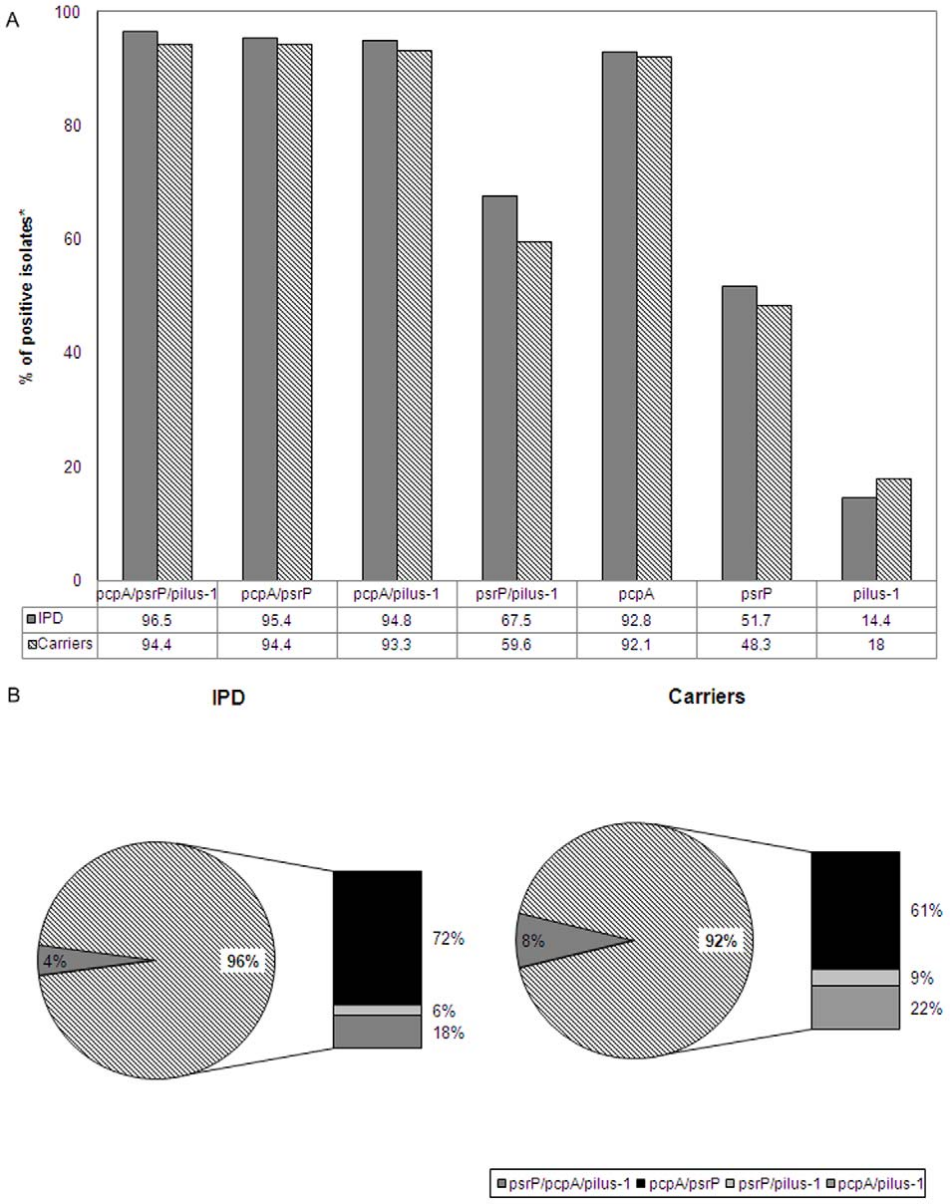
Prevalence of *pcpA, psrP* and pilus-1. (A) Prevalence for *pcpA, psrP* and pilus-1 alone and for their combinations (isolates with al least one of the three combinations) in 458 pneumococcal isolates of patients with invasive pneumococcal disease (IPD) and in 89 pneumococcal isolates of healthy nasopharyngeal carriers. (B) Prevalence of strains that carry all three proteins, and two of possible protein combinations including *pcpA* and *psrP, psrP* and pilus-1, *pcpA* and pilus-1 among pneumococcal isolates of patients with IPD and healthy nasopharyngeal carriers.

### Statistical Analysis

Statistical analysis was performed with the PASW software package (version 17.0). Continuous variables were compared using the t test (for approximately normally distributed data) or the Mann-Whitney U test (for skewed data) and described as mean values and standard deviations or median and interquartile range P25–P75 (IQR) according to the presence of normal distribution. Chi-square test or Fisher’s exact test (two-tailed) was used to compare categorical variables. Comparison between groups was performed by Kruskal-Wallis test. Statistical significance was set at a *P* value of <0.05.

**Figure 2:**
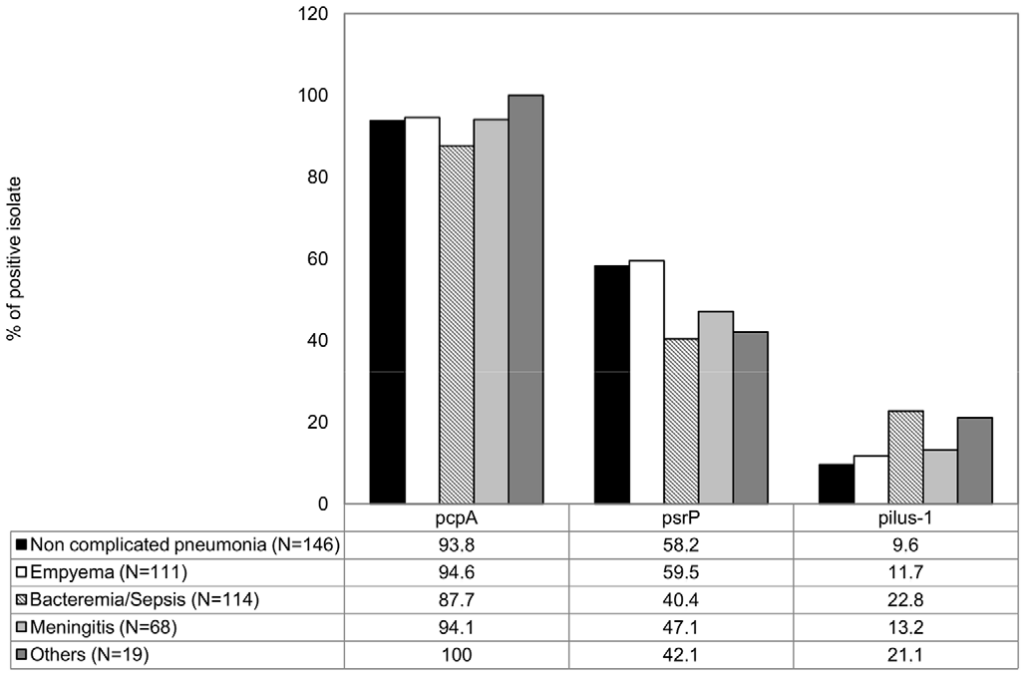
Prevalence of *pcpA, psrP* and pilus-1 according to clinical syndrome among pneumococcal invasive isolates.

**Table 1:** Prevalence of *pcpA, psrP* and pilus-1 according to antimicrobial susceptibility.

Antimicrobial agent			pcpA		psrP		pilus-1	
MIC	Isolates	%	%Positive	P	%Positive	P	%Positive	P
**Penicillin**								
≤0.06	409	75.3	90.2	<.000	62.6	<.000	8.8	<.001
≥0.12	134	24.7	100		17.9		34.3	
**Cefotaxime**								
≤0.5	482	88.8	91.7	0.01	56.4	<.000	9.3	<.001
≥1	61	11.2	100		13.1		60.7	
**Erythromicine**								
≤0.25	415	76.4	90.6	0.001	58.3	<.000	10.8	<.001
≥0.5	128	23.6	99.2		29.7		28.9	
Tetracycline*								
≤2	409	75.9	91.4	0.07	58.9	<.000	11.2	0.001
≥4	130	24.1	96.2		28.5		27.7	
Chloramphenicol**								
≤4	515	95.2	92.8	0.4	50.7	0.02	15.7	0.09
≥8	26	4.8	88.5		73.1		3.8	

The study was non-available in four* and six** isolates.

## Results

### Strain Properties

Of the total 461 pediatric invasive pneumococcal isolates in our library, 3 of them could not be recovered from stocks and were thereby excluded from the study. As such, we examined a total of 458 invasive pneumococcal isolates and 89 nasopharyngeal pneumococcal isolates among children (total  = 547 strains).

The clinical syndromes were: pneumonia 257 (111 of them with empyema), bacteremia 114, meningitis 68, arthritis 13, appendicitis 4, pericarditis 1 and peritonitis 1.

The most frequent serotypes detected among invasive isolates were serotype 1 (n = 134), 19A (n = 84), 7F (n = 35), 5 (n = 34) and 14 (n = 19). Among carriers the most frequent serotypes were 19A (n = 9), 6A (n = 9), 19F (n = 7), 15B (n = 6) and 23B (n = 6).

Among IPD isolates, the prevalence of serotypes included in the commercialized conjugate vaccines PCV7, PCV10 and PCV13 were 14.2% (65 isolates), 58.3% (267 isolates) and 83.6% (383 isolates) respectively. The prevalence of serotypes included in the three vaccines among isolates from the nasopharynx of healthy carriers was 23.6% (21 isolates), 27% (24 isolates) and 50.6% (45 isolates).

With respect to clonal properties the most frequent clonotypes among invasive isolates were ST306 (n = 107), ST191 (n = 31), ST1223 (n = 25), ST304 (n = 22), ST276 (n = 17). A high variety of clonotypes were detected in carriers (56 different clonotypes in 89 strains); the most frequent being ST2372 (n = 5), ST97 (n = 4), ST42 (n = 3), ST63 (n = 3), ST180 (n = 3), ST838 (n = 3) and ST2690 (n = 3). Finally, antibiotic susceptibility study was available in 543 of the 547 strains with 134 (24.5%) having diminished penicillin susceptibility (MIC ≥0.12). The percentage of isolates with diminished penicillin susceptibility was 23.4% (107 of 454) among invasive isolates and 30.3% (27 of 89) among carriers.

### Overall Prevalence of PcpA, *PsrP* and Pilus-1

The individual prevalence of *pcpA*, *psrP*, and Pilus-1 in the 547 strains of our collection were 92.7%, 51.2% and 15% without significant differences occurring between invasive and carrier isolates: for *pcpA* 92.8% vs. 92.1%; *P* = 0.8, for *psrP* 51.7% vs. 48.3%; *P* = 0.5 and for pilus-1 14.4% vs. 18%; *P* = 0.3, respectively. Given the high prevalence of *pcpA* the potential coverage with at least one protein of a multivalent vaccine including these three candidates would be high: 96.5% among invasive isolates (442 of 458 isolates) and 94.4% among carriers (84 of 89 isolates). Figure 1A shows the prevalence for each protein alone and for at least 1 of the proteins in the specific combinations (PcpA/PsrP/Pilus-1, PcpA/PsrP, PcpA/Pilus-1 and PsrP/Pilus-1). Notably, in Figure 1B, we show that 96% of the invasive isolates carried at least two of the three proteins, whereas 92% of the carrier isolates did the same. Likewise, 6% of isolates carried all 3 proteins, (4% and 8% of the invasive and carrier isolates, respectively). Thus, the majority of individuals immunized with a vaccine composed of these three antigens would have antibodies for at least 2 of these 3 proteins.

### Prevalence Based on Clinical Symptom and Antibiotic Resistance

The prevalence of *pcpA* among all strains was too high to have any correlation with any clinical condition. In contrast, the prevalence of *psrP* was significantly higher in patients with non complicated pneumonia (58.2; %*P*<.001) or empyema (59.5%; *P*<.001) than in children with bacteremia (40.4%). Inversely, the prevalence of pilus-1 was greater in patients with bacteremia than in patients with non-complicated (22.8% vs. 9.6%; P = 0.005) and complicated pneumonia (11.7%; P = 0.04) (Figure 2). We also observed significant differences in the prevalence of *psrP* and pilus-1 according to susceptibility for different antimicrobials (Table 1). Overall *psrP* was significantly more frequently detected in penicillin, cefotaxime, erythromycin and tetracycline susceptible isolates while pilus-1 and, to a modest level *pcpA*, were more frequently detected in isolates non susceptible to these antimicrobials. In contrast, *psrP* was significantly more frequently detected in chloramphenicol non-susceptible isolates.

### Prevalence of *pcpA*, *psrP* and Pilus-1 According to Serotype and Clonotype

Prevalence of these proteins was strongly associated with specific serotype and clonotypes. Table 2 shows significant differences in the prevalence of *pcpA*, *psrP* and pilus-1 according to serotype. *pcpA* is highly prevalent in almost all serotypes, the exception being serotype 3. *pcpA* was only detected in 7 of 21 isolates of serotype 3 (33.3%) vs. 500 of 526 non serotype 3 isolates (95.1%; P<.001). Interestingly, for certain serotypes the prevalence of *psrP* was high but occurred with an absence of pilus-1 or vice versa. For example, the prevalence of *psrP* among 136 strains tested of serotype 1 was 80.1% (109 isolates) but pilus-1 was not detected in any strain of serotype 1. This observation was also detected for serotype 5 where *psrP* was detected in 88.2% of the 34 strains but Pilus-1 was absent. In contrast, for serotypes 14 or 6B the prevalence of *psrP* was significantly lower than the prevalence of pilus-1 (5.3% vs. 84.2% among serotype 14 isolates (n = 19) and 35.7% vs. 57.1% among serotype 6B isolates (n = 14). Other serotypes without pilus-1 included serotype 7F (none of 36 strains) and serotype 3 (none of 21 strains). *psrP* was also very low in these serotypes (11.1% for serotype 7F and 9.5% for serotype 3). In fact, of all 547 strains tested, only 4.2%, tested positive for both *psrP* and pilus-1.

**Table 2:** Prevalence of *pcpA,psrP* and Pilus-1 according to serotype of isolates.

Serotype	Isolates	*pcpA* Pos	%	*psrP* Pos	%	pilus Pos	%
Overall	547	507	92.7	280	51.2	82	15.0
1	136	132	97.1	109	80.1	0	0.0
19A	93	90	96.8	44	47.3	26	28.0
7F	36	36	100.0	4	11.1	0	0.0
5	34	28	82.4	30	88.2	0	0.0
6A	22	21	95.5	11	50.0	4	18.2
3	21	7	33.3	2	9.5	0	0.0
19F	20	16	80.0	13	65.0	6	30.0
14	19	19	100.0	1	5.3	16	84.2
6B	14	14	100.0	5	35.7	8	57.1
15B	13	13	100.0	10	76.9	2	15.4
9V	12	12	100.0	3	25.0	9	75.0
23B	12	12	100.0	1	8.3	0	0.0
24F	10	10	100.0	1	10.0	0	0.0
23F	10	10	100.0	1	10.0	0	0.0
10A	9	9	100.0	2	22.2	1	11.1
23A	6	6	100.0	4	66.7	0	0.0
18C	6	6	100.0	5	83.3	0	0.0
15C	6	6	100.0	5	83.3	1	16.7
38	6	3	50.0	2	33.3	2	33.3
21	5	5	100.0	3	60.0	0	0.0
4	5	3	60.0	4	80.0	4	80.0
15A	4	4	100.0	1	25.0	0	0.0
24	4	4	100.0	2	50.0	0	0.0
35B	3	3	100.0	1	33.3	1	33.3
22F	3	3	100.0	3	100.0	0	0.0
16F	3	3	100.0	3	100.0	0	0.0
12F	3	3	100.0	0	0.0	0	0.0
9N	2	2	100.0	1	50.0	0	0.0
37	2	2	100.0	0	0.0	0	0.0
34	2	1	50.0	0	0.0	1	50.0
31	2	2	100.0	0	0.0	0	0.0
29	2	2	100.0	1	50.0	0	0.0
28	2	1	50.0	0	0.0	0	0.0
27	2	1	50.0	0	0.0	0	0.0
22	2	2	100.0	1	50.0	0	0.0
16	2	2	100.0	2	100.0	0	0.0
6C	1	1	100.0	0	0.0	0	0.0
35F	1	1	100.0	1	100.0	0	0.0
33F	1	1	100.0	0	0.0	0	0.0
24B	1	1	100.0	0	0.0	0	0.0
17F	1	1	100.0	1	100.0	0	0.0
11A	1	1	100.0	0	0.0	0	0.0
47	1	1	100.0	0	0.0	1	100.0
39	1	1	100.0	1	100.0	0	0.0
17	1	1	100.0	0	0.0	0	0.0
13	1	1	100.0	1	100.0	0	0.0
11	1	1	100.0	1	100.0	0	0.0
10	1	1	100.0	0	0.0	0	0.0
8	1	1	100.0	0	0.0	0	0.0
2	1	1	100.0	0	0.0	0	0.0

Pos: positive detection.

**Figure 3:**
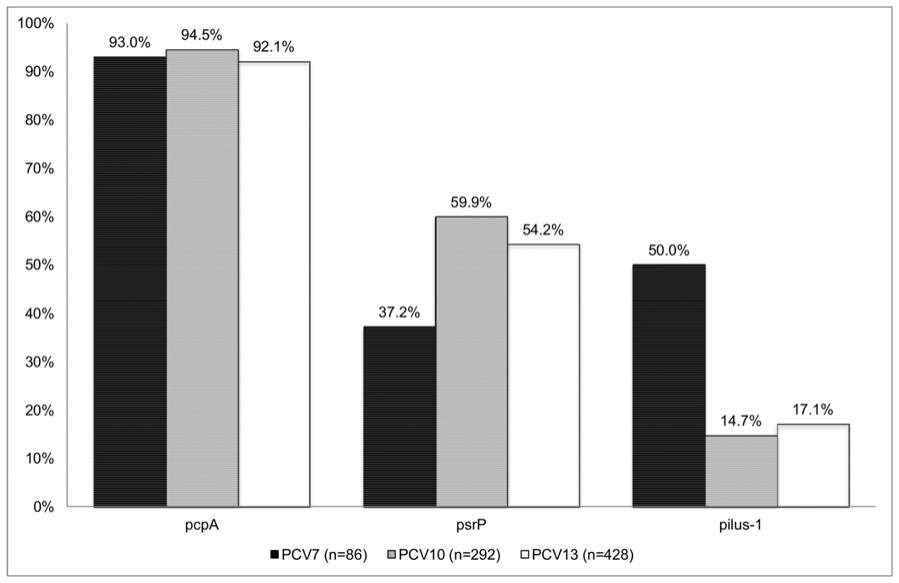
Prevalence of *pcpA, psrP* and pilus-1 according to serotypes within the commercialized conjugate vaccines.

Using the designation of serotypes having high or low attack rate [21], [22] *psrP* was significantly more frequent in serotypes categorized as having high attack rate (those less apt to be detected in carriage than in disease) than in serotypes categorized as low attack rate (those less apt to be detected in disease than in carriage) (58.7% vs. 39.1%; P<.001). *pcpA* was also more frequently detected in serotypes with high attack rate (95.6% vs. 87.5%; *P* = 0.01). Pilus-1 distribution was similar in high and low attack rate serotypes (16.1% vs. 13.6%; *P* = 0.4). Considering only penicillin susceptible isolates, the prevalence of *psrP* between high and low attack rate serotypes was different (72.3% vs. 44.9%; P<.001). The distribution of *pcpA* among these susceptible isolates was also higher in high attack rate serotypes vs. low attack rate serotypes (94.3% vs. 81.9%; P = 0.01). Among penicillin susceptible isolates, the prevalence of Pilus-1 was higher in those expressing serotypes that are less apt to be detected in disease than in carriage (13.4% vs. 6.4%; P = 0.02). Figure 3 shows the prevalence of PcpA, PsrP and Pilus-1 according to serotypes within the commercialized conjugate vaccines. Pilus-1 was more frequent detected among PCV7 serotypes vs. non PCV7 serotype 50% vs. 8.5%; P<001). In contrast, *psrP* was more frequent detected among non PCV7 isolates vs PCV7 isolates (53.8% vs. 37.2%; P = 0.005).

Finally, we observed stark and significant differences in prevalence of these proteins according to clonotype among isolates expressing the same serotype (Table 3). *psrP* was detected in almost all ST306 (106 of 109 isolates; 97.2%) while practically in none of the isolates with ST304 (1 of 22 isolates; 4.5%). Pilus-1 was totally absent in these clonotypes. The same phenomenon was observed for the penicillin susceptible clone ST1201: all isolates with this clone (n = 19) have *psrP*, while none have Pilus-1. The opposite was observed for multiresistant clone ST320, which all (n = 16) have pilus-1 yet lack *psrP*. Even in *pcpA*, which has a high prevalence within the entire collection, significant differences according to clonotype were detected in strains expressing the same serotype. For example, among isolates expressing serotype 3, *pcpA* was detected in 100% of strains with ST260, ST1220, ST1377 or ST2590 (6 isolates) while only in 6.6% of ST180 (1 of 15 isolates).

**Table 3:** Prevalence of *pcpA. psrP* and pilus-1 according to clonotypes (ST) detected in the study.

			pcpA		psrP		pilus-1	
ST	Isolates	Serotype	Positive	% Positive	Positive	% Positive	Positive	% Positive
306	109	1 (n = 109)	107	98.2	106	97.2	0	0.0
191	32	7F (n = 32)	32	100.0	3	9.4	0	0.0
1223	25	5 (n = 25)	23	92.0	21	84.0	0	0.0
304	22	1 (n = 22)	22	100.0	1	4.5	0	0.0
1201	19	19A (n = 19)	18	94.7	19	100.0	0	0.0
276	18	19A (n = 18)	18	100.0	3	16.7	0	0.0
320	16	19A (n = 16)	16	100.0	0	0.0	16	100.0
180	15	3 (n = 15)	1	6.7	1	6.7	0	0.0
156	13	14 (n = 13)	13	100.0	0	0.0	13	100.0
2013	13	19A (n = 13)	13	100.0	2	15.4	0	0.0
2372	12	23B (n = 10)	10	83.4	1	8.3	0	0.0
		19A (n = 1)	1	8.3	1	8.3	1	8.3
		23F (n = 1)	1	8.3	0	0	0	0
97	11	10A (n = 11)	11	100.0	2	18.2	1	9.1
289	8	5 (n = 8)	4	50.0	8	100.0	0	0.0
63	7	15A (n = 4)	4	57.1	1	14.3	0	0.0
		15B (n = 1)	1	14.3	1	14.3	0	0.0
		15C (n = 1)	1	14.3	0	0.0	0	0.0
		38 (n = 1)	1	14.3	1	14.3	0	0.0
4677	6	24F (n = 6)	6	100.0	0	0.0	0	0.0
2100	6	19F (n = 6)	6	100.0	1	16.7	0	0.0
1167	6	19F (n = 5)	1	16.7	5	83.3	4	66.6
		19A (n = 1)	0	0.0	1	16.7	1	16.7
838	6	9V (n = 6)	6	100.0	0	0.0	6	100.0
230	6	24F (n = 3)	3	50.0	0	0.0	0	0.0
		24 (n = 2)	2	33.3	0	0.0	0	0.0
		24B (n = 1)	1	16.7	0	0.0	0	0.0
202	6	19A (n = 6)	5	83.3	2	33.3	5	83.3
113	6	18C (n = 6)	6	100.0	5	83.3	0	0.0
199	5	19A (n = 4)	4	80.0	4	80.0	0	0.0
		15B (n = 1)	1	20.0	1	20.0	0	0.0
42	5	23A (n = 5)	5	100.0	4	80.0	0	0.0
1262	4	15B (n = 2)	2	50.0	2	50.0	0	0.0
		15C (n = 2)	2	50.0	2	50.0	0	0.0
433	4	22 (n = 1)	1	25.0	0	0.0	0	0.0
		22F (n = 1)	1	25.0	1	25.0	0	0.0
		19A (n = 1)	1	25.0	0	0.0	0	0.0
		28 (n = 1)	1	25.0	0	0.0	0	0.0
416	4	19A (n = 4)	4	100.0	4	100.0	1	25.0
386	4	6B (n = 4)	4	100.0	1	25.0	2	50.0
90	4	6A (n = 2)	1	25.0	0	0.0	2	50.0
		6B (n = 2)	2	50.0	0	0.0	2	50.0
81	4	19A (n = 2)	2	50.0	2	50.0	0	0.0
		19F (n = 1)	1	25.0	1	25.0	0	0.0
		23F (n = 1)	1	25.0	1	25.0	0	0.0
30	4	16 (n = 2)	2	50.0	2	50.0	0	0.0
		16F (n = 2)	2	50.0	2	50.0	0	0.0
2690	3	29 (n = 2)	2	66.7	1	33.3	0	0.0
		21 (n = 1)	1	33.3	0	0.0	0	0.0
1684	3	31 (n = 2)	2	66.7	0	0.0	0	0.0
		1 (n = 1)	1	33.3	0	0.0	0	0.0
1143	3	6A (n = 3)	3	100.0	3	100.0	1	33.3
310	3	38 (n = 2)	0	0.0	0	0.0	2	66.7
		34 (n = 1)	0	0.0	0	0.0	1	33.3
280	3	9V (n = 2)	2	66.7	2	66.7	0	0.0
		9N (n = 1)	1	33.3	1	33.3	0	0.0
224	3	6A (n = 3)	3	100.0	0	0.0	0	0.0
193	3	21 (n = 2)	2	66.7	2	66.7	0	0.0
		15B (n = 1)	1	33.3	1	33.3	0	0.0
101	3	15C (n = 2)	2	66.7	2	66.7	0	0.0
		15B (n = 1)	1	33.3	0	0.0	0	0.0
72	3	24 (n = 2)	2	66.7	2	66.7	0	0.0
		24F (n = 1)	1	33.3	1	33.3	0	0.0

Other ST detected with 2 isolates each: ST62, ST109, ST162, ST177, ST338, ST393, ST439, ST447, ST558, ST989, ST1011, ST1220, ST1377, ST1624, ST1692, ST2611, ST2948, ST4310, ST4828, ST5223, and ST5740.

1 isolate each: ST9, ST66, ST88, ST94, ST110, ST124, ST143, ST176, ST179, ST205, ST217, *S*T228, *S*T245, *S*T260, *S*T274, *S*T311, *S*T315, *S*T327, *S*T343, *S*T392, *S*T404, *S*T425, *S*T446, *S*T450, *S*T460, *S*T494, *S*T557, *S*T614, *S*T876, *S*T994, *S*T1012, *S*T1064, *S*T1264, *S*T1475, *S*T1504, *S*T1577, *S*T1589, *S*T1611, ST1664, *S*T1844, *S*T1848, *S*T2319, *S*T2333, *S*T2376, *S*T2377, *S*T2467, *S*T2557, *S*T2590, *S*T2592, *S*T2594, *S*T2595, *S*T2618, *S*T2946, *S*T2947, *S*T2949, *S*T3254, *S*T3259, *S*T3436, *S*T3437, *S*T3438, ST3490, *S*T3609, *S*T3787, *S*T4306, *S*T4676, *S*T4796, *S*T4826, *S*T4832, *S*T4834, *S*T5224, ST5741, *S*T5825, *S*T5829, *S*T6006, *S*T6040, *S*T6394, *S*T6518 and *S*T6519.

## Discussion

Among IPD isolates, the prevalence of disease caused by serotypes included in the commercialized conjugate vaccines increased from 14.2% in PCV7 to 83.6% in PCV13. In contrast, the overall prevalence of serotypes included in PCV13 in nasopharynx was only 50.6%. Thus, even though the newly introduced PCV13 vaccine had robust coverage against disease, its intermediate coverage of the current colonizing serotypes leaves open the possibility of serotype replacement by current invasive clones or continuing serotype shift. In the same way that an indirect effect of PCV7 preventing disease in adults and non-vaccinated children had been observed [5], [6], it is expected indirect protection offered by herd immunity using multivalent pneumococcal protein vaccines [26], [27].

*PcpA* was highly prevalent in our collection, suggesting that it is a conserved pneumococcal component. While previous studies, including our own, have examined the prevalence of *psrP* or pilus-1 alone among clinical isolates [28]–[31], to our knowledge no information exists on the prevalence of *pcpA*. As indicated PcpA is an adhesin, and immunization with recombinant protein has been demonstrated to reduce the number of bacteria in the lungs of mice challenged with *S. pneumoniae* and to increase survival time in a mouse sepsis model following intraperitoneal challenge [19]. Most recently, PcpA has been shown to be required for *in vitro* biofilm formation [32], upregulated in response to Zn(2+) [33], and capable of eliciting antibodies during human nasopharyngeal colonization and acute otitis media [34], but not during bacteremia in infants [35]. Our finding that *pcpA* was present in 500 of the 526 serotypes, excluding serotype 3 isolates, underlines the importance of this protein for pneumococcal biology and strongly supports its inclusion in any protein vaccine.

Surprisingly, *pcpA* was only present in 7 of the 21 serotype 3 isolates tested. The absence of adhesins in serotype 3 isolates is not unprecedented; Choline binding protein A (CbpA; also known as PspC), which binds to both polymeric immunoglobulin receptor and laminin receptor, and has been implicated in biofilm formation, has a low prevalence within serotype 3 isolates [36]. Serotype 3 isolates are distinct from most other pneumococcal serotypes in that they are exceedingly encapsulated, and therefore appear highly mucoid on blood agar plates. The absence of these adhesins and a distinct clinical profile suggest that serotype 3 isolates might have a pathogenesis dissimilar to other pneumococcal isolates, as numerous studies indicate that capsular polysaccharide inhibits bacterial adhesion, and serotype 3 isolates are frequently associated with necrotizing pneumonia. This suggests that a distinct protein vaccine formulation would be required for protection against serotype 3-mediated disease. This notion is supported by studies in experimentally infected mice, where a serotype 3 clinical isolate remained in the lungs but replicated to high titers, whereas clinical isolates of serotype 2 and 4 replicated to lower titers but caused disseminated disease [37].

PsrP is both an intraspecies and interspecies adhesin, mediating attachment to Keratin 10 on lung cells and promoting the presence of bacterial aggregates *in vivo* and biofilm formation *in vitro* [38]. Pilus also functions as an adhesin, having been demonstrated to mediate attachment to laminin and may also contribute to the invasiveness of strains [39].

Importantly, considerable evidence indicates that immunization of mice with either the basic region domain of PsrP or with individual components of Pilus-1 mediates protection [16], [40]. Using Real-Time PCR, we detected *psrP* in 51.2% of all clinical isolates, whereas we detected pilus-1 in 15% of all isolates. This was consistent with a past study where the prevalence of *psrP* in clinical isolates was found to be 52.4% and with studies of numerous other investigators where the prevalence of pilus-1 in clinical isolates was found to be between 10–30% [30], [31], [41], [42].

Our study expands on these past studies by providing the prevalence of these candidate vaccine antigens simultaneously. There by assessing the potential coverage of a multivalent vaccine composed of *pcpA*, *psrP* and pilus-1. In all, 96% of the strains examined carried at least 1 of these proteins, 96% carried 2, and 6% carried all 3. Our analysis determined that *psrP* and pilus-1 have a negative correlation in multiple serotypes raising the possibility that *psrP* and pilus-1 may have redundant roles, or that their production might be metabolically expensive and that an individual strain cannot support production of both of these extremely large proteins. Briefly, PsrP is a glycosylated surface protein that separates at a molecular weight >2000 kDa, whereas Pilus-1 is primarily composed of multiple repeats of the subunit RrgB. Both extend beyond the bacterial capsule to mediate adhesion. Interestingly, our study shows that *psrP* was found significantly among serotypes that are less apt to be detected in carriage than in disease, while Pilus-1 was not associated with these virulent serotypes. These data could suggest that PsrP is in part responsible for the increased virulence of high attack rate serotypes. Along this line, it is known that variation in virulence exists among isolates of the same serotype, due to the contribution of serotype-independent factors associated with clonal type [43]. The variability of the prevalence of *pcpA*, *psrP* and pilus-1 according to clonal type in strains expressing the same serotype confirms that the presence of these factors appears to be a clonal property. This fact has been reported for Pilus-1 by other authors [41].

Antibiotic resistance was associated with the presence of pilus-1 and showed a negative correlation with *psrP*. The association of pilus-1 with antibiotic resistance has been reported previously, but the reasons for this association are not clear. It could be that the *rrlA* islet and specific resistance genes might be recombined together. Moschioni et al. suggest that pilus aid in adhesion during colonization of the nasopharynx and that pilus expressing strains could be selected as a result of antibiotic treatment [44]. The reason for negative association of *psrP* with resistant strains is unknown. Interestingly, *psrP* had greater correlation with strains isolated from individuals with pneumonia, both uncomplicated and complicated, whereas Pilus-1 had a predilection for strains associated with bacteremia. This observation is consistent with the known roles of PsrP as a lung cell adhesin and Pilus-1 as a mediator of invasive disease [17].

A limitation of the study is that the absence or presence of these genes/proteins is based on PCR results of wellknown and published genes [15], [18], [44] but potential primer divergence could implied that a PCR negative result is not necessary equivalent of the absence of the protein and viceversa.

In summary, our results indicate that *pcpA* is highly prevalent and its addition to a multivalent pneumococcal protein vaccine would result in considerable coverage. In contrast, *psrP* and pilus-1 have less robust individual coverage but, since *psrP* is present in high attack rate strains and pilus-1 in antibiotic resistant strains, could be added in an effort to reduce the likelihood of disease. The inverse correlation of these proteins suggests that they could be paired as part of a multi-valent vaccine to compensate for each other. This notion is highlighted by the fact that 96% of all strains carried *pcpA* and either *psrP* or pilus 1. Future studies are planned to determine the protective efficacy of this trivalent vaccine against invasive disease caused by multiple clinical isolates.
